# Metabolic cross-talk within the bone marrow milieu: focus on multiple myeloma

**DOI:** 10.1186/s40164-022-00303-z

**Published:** 2022-09-01

**Authors:** Inge Oudaert, Arne Van der Vreken, Anke Maes, Elke De Bruyne, Kim De Veirman, Karin Vanderkerken, Eline Menu

**Affiliations:** Department of Hematology and Immunology, Myeloma Center Brussels, Vrije Universiteit Brussel, 1090 Brussels, Belgium

**Keywords:** Multiple myeloma, Metabolism, Glycolysis, Oxidative phosphorylation, Glutamine metabolism, Lipid metabolism, Glucose metabolism, Lactate metabolism, Hypoxia, Bone marrow microenvironment

## Abstract

Cancer cells are well-known for their capacity to adapt their metabolism to their increasing energy demands which is necessary for tumor progression. This is no different for Multiple Myeloma (MM), a hematological cancer which develops in the bone marrow (BM), whereby the malignant plasma cells accumulate and impair normal BM functions. It has become clear that the hypoxic BM environment contributes to metabolic rewiring of the MM cells, including changes in metabolite levels, increased/decreased activity of metabolic enzymes and metabolic shifts. These adaptations will lead to a pro-tumoral environment stimulating MM growth and drug resistance In this review, we discuss the identified metabolic changes in MM and the BM microenvironment and summarize how these identified changes have been targeted (by inhibitors, genetic approaches or deprivation studies) in order to block MM progression and survival.

## Introduction

Multiple myeloma (MM) is a hematological malignancy characterized by the accumulation of monoclonal plasma cells in the bone marrow (BM). It is the second most frequent hematological cancer, and globally affects about 750,000 people [1]. Around 35,000 Europeans are newly diagnosed every year, but this incidence is expected to increase to 43,000 by 2030 [2]. Patients are typically diagnosed around the age of 72 years, and median survival is 8–9 years [3]. The most prominent symptoms include hypercalcemia, renal failure, anemia and osteolytic lesions [4, 5], also known as the CRAB symptoms. Diagnosis is made by measuring the levels of monoclonal proteins in urine or serum (M-spike), by BM aspirate (≥ 60% of clonal plasma cells) and at least one focal lesion on magnetic resonance imaging [6]. Patients suffering from MM evolve from precancerous but asymptomatic stages called monoclonal gammopathy of undetermined significance (MGUS) and smoldering MM (SMM) [7, 8].

Over the past decades, numerous new therapies for myeloma have been developed. Therapy options can be divided into two main groups: therapies for transplant-eligible and non-eligible patients. Patients eligible for autologous stem cell transplantation will typically receive an induction therapy with the proteasome inhibitor bortezomib, immunomodulating agent lenalidomide and steroid dexamethasone (VRd) for 3–4 cycles, before stem cells are harvested [9]. When contra-indicated, bortezomib can be combined with the immunomodulating agents thalidomide and dexamethasone (VTd) or with cyclophosphamide and dexamethasone (VCd) [9]. In case of non-eligibility, either due to age or other comorbidities, patients are treated with VRd. After 8–12 weeks of VRd treatment, patients will receive maintenance therapy consisting of lenalidomide [9]. More recently, several new drugs, including monoclonal antibodies daratumumab and elotuzumab, as well as BCMA-targeted CAR-T cell therapy have been FDA-approved for treatment of newly diagnosed (NDMM) and/or relapsed and refractory patients (RRMM). Although these treatment strategies initially work, most patients will eventually relapse due to drug resistance, rendering myeloma an incurable cancer [9].

Drug resistance can be caused by either intrinsic or extrinsic mechanisms [10–13]. Intrinsically, myeloma cells can be resistant to drugs due to mutations or translocations (p-glycoproteins, IGF1-IGF1 receptor) [14, 15]. Other intrinsic mechanisms causing drug resistance are metabolic changes and epigenetic modifications. Extrinsically, drug resistance is mediated by the BM micro-environment and can be subdivided into two groups: soluble factor-mediated drug resistance (release of cytokines, chemokines, growth factors and exosomes) and cell adhesion-mediated drug resistance (adhesion between MM cells and stromal cells and fibronectin via β1-integrins) [11, 12, 16].

The BM microenvironment plays an important role in drug resistance and consists out of a cellular (hematopoietic cells vs. non-hematopoietic cells) and non-cellular compartment. The hematopoietic compartment is made up by the MM cells, T and B lymphocytes, myeloid cells, natural killer cells, macrophages, monocytes, dendritic cells, platelets, osteoclasts, erythrocytes and megakaryocytes, while the non-hematopoietic cells consist of BM stromal cells (BMSCs), vascular endothelial cells, fibroblasts, osteoblasts and adipocytes [17]. Extracellular matrix proteins including laminin, collagen and fibronectin; adhesion molecules; cytokines and growth factors make up the non-cellular compartment [4, 16, 18]. The BM microenvironment supports the growth and survival of MM cells. In turn, MM cells will influence the BM niche to create a pro-tumoral environment, adapting the structure and composition of the BM environment to fully support the MM cells during progression [19, 20]. MM cells strongly adhere to BMSCs via several molecules, including but not limited to CD44, very late antigen (VLA)-4 and 5, CD40/CD40L and intercellular adhesion molecule 5 (ICAM5) [19, 21].

Although several advancements have been made in the last decade regarding cancer therapies, one of the biggest challenges of the twenty-first century remains the development of drug resistance. Therefore, new therapeutic targets continue to be explored. In recent years, metabolomics has gained a lot of attention in the search for new cancer therapies. Cancer cells are able to adapt to unfavorable conditions by changing their metabolism. For MM, many of these metabolic changes are induced by the hypoxic niche.

In this review, we will focus on the metabolic changes that have been identified in MM and its environment. First, an overview of the already known metabolic alterations in MM cells, how they contribute to drug resistance and their targetability (by inhibitors, genetic approaches or deprivation studies) will be discussed. Second, we will describe how the complex interplay between the MM cells and other components of the BM microenvironment contributes to metabolic alterations in MM cells.

## General and altered cancer cell metabolism

**Fig. 1:**
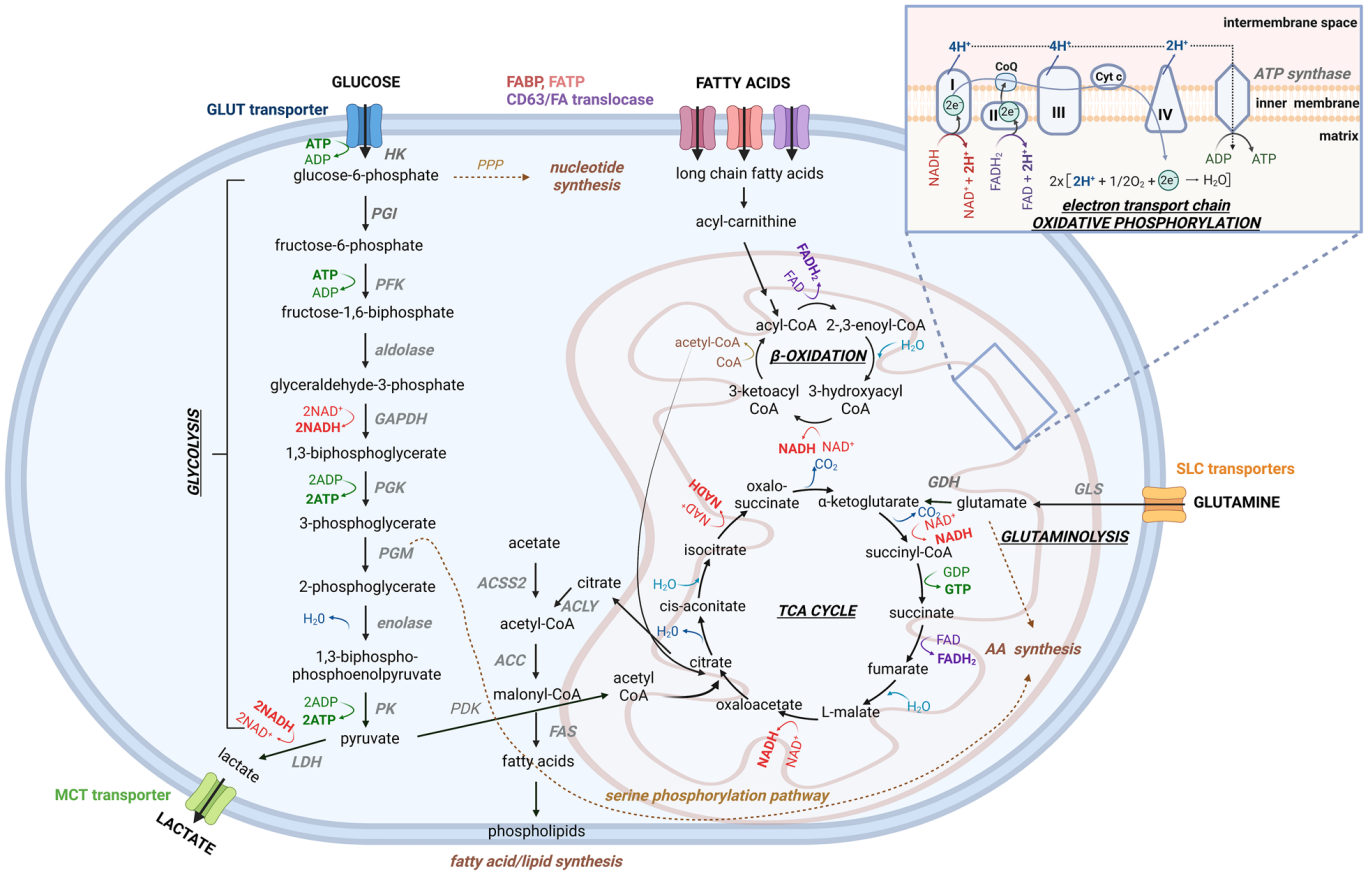
Overview of the most important metabolic processes to generate energy in human cells: glycolysis, mitochondrial respiration, glutaminolysis and fatty acid oxidation. (1) During glycolysis, glucose is taken up into the cells by glucose transporters (GLUT) and converted into pyruvate by several enzymatic processes. Finally, pyruvate is transformed into lactate, which is transported out of the cell by monocarboxylate transporters (MCT). Pyruvate can also be converted to acetyl-CoA by pyruvate dehydrogenase kinase (PDK), which then enters the tricarboxylic acid (TCA) cycle for mitochondrial respiration (2). During this multistep process, NADH and FADH_2_ are produced. Both molecules are necessary to complete the oxidative phosphorylation and drive the electron transport chain, resulting in the production of ATP. 3) During glutaminolysis, glutamine is taken up into the cells by several soluble carrier (SLC) transporters including large amino acid transporter 1 (LAT1, also known as SLC7A5), alanine, serine, cysteine transporter 2 (ASCT2 also known as SLC1A5) and sodium-coupled neutral acid transporter 1 (SNAT1 also known as SLC38A1). Once glutamine is intracellularly present, glutamine is transformed into glutamine and α-ketoglutarate, where it enters the TCA cycle. 4) Finally, fatty acids are taken up into the cell by fatty acid binding protein (FABP), fatty acid transporter protein (FATP) and CD63/fatty acid translocase, where fatty acids are converted into long chain fatty acids and acyl-carnithine, followed by translocation to the inner mitochondrial membrane, where acyl-carnithine is transformed into acyl-CoA and carnithine. During β-oxidation, acyl-CoA undergoes several reactions, generating NADH, FADH_2_ (both necessary for the electron transport chain during oxidative phosphorylation) and acyl-CoA and acetyl-CoA (necessary for TCA cycle). Enzymes are shown in grey. *HK:* hexokinase, *PGI: *phosphoglucose isomerase, *PFK: *phosphofructokinase, *GAPDH:* glyceraldehyde-3-phosphate dehydrogenase, *PGK: *phosphoglycerate kinase, *PGM: *phosphoglyceromutase, *PK:* pyruvate kinase, *LDH:* lactate dehydrogenase, *ACLY:* ATP citrate lyase, *ACC:* acetyl-CoA carboxylase, *PDK:* pyruvate dehydrogenase kinase, *AA: *amino acid, *GLS:* glutaminase, *GDH:* glutamate dehydrogenase, *FAS: *fatty acid synthase, *FA:* fatty acid, *ADP: *adenosine diphosphate, *ATP:* adenosine triphosphate, *NAD: *nicotinamide adenine dinucleotide, *FAD: *flavin adenine dinucleotide, *PPP: *pentose phosphate pathway, *GDP: *guanine diphosphate, *GTP: *guanine triphosphate, *Cyt C: *cytochrome *C*, *CoQ: *coenzyme Q_10_. Created with BioRender.com

Every cell in the body needs to take up nutrients in order to generate energy for proliferation, migration and differentiation. The most universal nutrients for cell growth are glucose and glutamine. Practically all cells use glycolysis, the breakdown of glucose into two moles of pyruvate and two moles of adenosine triphosphate (ATP), as the main metabolic pathway to generate energy. The import of glucose is facilitated by several transporters: GLUT1 in erythrocytes and vascular endothelium, GLUT2 in hepatocytes, β-cells, intestinal mucosa and renal cells, GLUT3 in proliferative cells and neurons and GLUT4 in skeletal and cardiac muscle and adipose tissue [22, 23]. Once glucose is taken up into the cell, the process of glycolysis can start. Hereby, glucose is broken down into glucose-6-phosphate, followed by subsequent conversion into fructose-6-phosphate, fructose-1,6-biphosphate, glyceraldehyde-3-phosphate, 1,3-biphosphoglycerate, 3-phosphoglycerate, 2-phosphoglycerate, 1,3-biphosphophosphoenolpyruvate (PEP), and finally pyruvate [23, 24] (Fig. 1). These conversions are made possible by the activity of several enzymes, including hexokinase 1 and 2 (HK1, HK2), phosphofructokinase (PFK) and pyruvate kinase M2 (PKM2). Next, pyruvate enters the matrix of the mitochondria, where the tricarboxylic acid (TCA) cycle, also known as the Krebs cycle, will start. Pyruvate will be transformed into acetyl-CoA, citrate, cis-aconitate, isocitrate, oxalo-succinate, α-ketoglutarate, succinyl-CoA, succinate, fumarate, l-malate and finally oxalo-acetate. This process generate one mole of guanine triphosphate (GTP), three moles of NADH and one mole of FADH_2_. Both NADH and FADH_2_ are established after NAD^+^ and FADH^+^ function as electron shuttles and capture the released electrons. Eventually, these electron shuttles translocate to the inner mitochondrial membrane as part of the electron transport chain to generate ATP [25, 26] (Fig. 1). This translocation also marks the start of oxidative phosphorylation. The energy-rich electrons from NADH and FADH_2_ will change O_2_ into H_2_O through a redox process (Fig. 1). This process releases energy, which is partly used to create a proton gradient, pumping protons from the matrix into the intermembrane space in the mitochondrion through protein complexes I, II and IV. This proton flux is a direct energy source to produce ATP, as protein complex V uses this energy to make ATP from adenosine diphosphate (ADP). Eventually, these subsequent metabolic processes generate 36 mol of ATP out of one mole of glucose in aerobic conditions [26]. In case of anaerobic conditions, glucose is transformed into lactate instead of pyruvate by lactate dehydrogenase A (LDHA), followed by excretion out of the cell through monocarboxylate transporters (MCT) [25].

Aside from glucose, glutamine is also a very important nutrient that many cancer cells depend on. Glutamine is a non-essential amino acid, involved in proliferation, apoptosis, cytokine production and differentiation [27]. Glutamine can be used as a building block for nucleic acids, lipids and proteins or as an extra energy source through glutamine-driven oxidative phosphorylation when converted into glutamate [28–30]. Glutamine is taken up into the cells by solute carrier family (SLC) transporters, followed by uptake into the mitochondria, where glutamine is transformed into glutamate by glutaminase and into alpha-ketoglutarate by glutamate dehydrogenases or aminotransferases before entering the TCA cycle [31] (Fig. 1). Aside from the mitochondrial uptake, glutamine can also be directly used as a source to produce nucleotides and/or uridine diphosphate N-acetylglucosamine for protein folding and protein trafficking [32, 33].

Fatty acid/lipid metabolism is a third metabolic route important for the generation of energy. Fatty acids consist out of long chains of hydrocarbons and a carboxylic acid group situated at the end of the hydrocarbon. Fatty acids are taken up into the cell by several proteins: the fatty acid binding protein (FABP) family, fatty acid transport protein (FATP) family and CD36/fatty acid translocase [34] (Fig. 1). During fatty acid oxidation, the fatty acids are converted into long-chain acyl-CoA, after which an acyl group is transported from fatty acyl-CoA to carnitine, forming acyl-carnitines. These molecules will then be transported into the mitochondrial matrix and the inner mitochondrial membrane, where acyl-carnitine is transformed into acyl-CoA and carnitine. Finally, acyl-CoA undergoes several reactions in the mitochondrial matrix during β-oxidation, releasing NADH and FADH_2_ and generating acyl-CoA and acetyl-CoA. Once acetyl-CoA is generated, this molecule will either enter the TCA cycle, where acetyl-CoA together with oxaloacetate will produce citrate through citrate synthase or be used during ketogenesis (Fig. 1). Fatty acid metabolism is very efficient, as each oxidation of acyl-CoA produces five mol of ATP [35, 36].

Lipids are built out of cholesterol, phospholipids (glycerol-based), and sphingolipids (ceramide-based) [37] and can be found in biological membranes, where they serve as building blocks. They also function as important metabolites that influence energy, structure and signaling [38–40]. Most lipids consist out of polymers of fatty acids. Lipids can be acquired by either nutritious uptake or through de novo lipogenesis. In normal healthy conditions, only hepatocytes and adipocytes are able to produce lipids. Other cell types generate lipids by either uptake of fatty acids out of the blood stream or through complex formation with proteins like low density lipoproteins [41]. De novo lipogenesis starts with uptake of glucose, followed by glycolysis and conversion into pyruvate and finally citrate through the TCA cycle. Citrate is then converted into acetyl-CoA, malonyl-CoA, palmitate and finally complex fatty acids or phospholipids (Fig. 1). Enzymes involved in the fatty acid synthesis pathway are ATP-citrate lyase, acetyl-CoA carboxylase and fatty acid synthase [42]. Several studies have shown that cancer cells show an increased uptake of lipids and an increased lipid synthesis as most of the enzymes involved in this process are upregulated or activated [36, 43, 44].

Although many different cancers rely on the energy supply from these metabolic processes, specific metabolic changes can vary from one cancer cell type to another [25, 45]. However, one of the most universal metabolic adaptions cancer cells make to comply with the increased energy demands is the change of mitochondrial oxidative phosphorylation to aerobic glycolysis. This change of preferred metabolism results in more conversion of glucose (and pyruvate) to lactate (instead of acetyl-CoA), a process referred to as the Warburg effect [46, 47]. Since this generates less ATP, the cancer cells need to increase the influx of glucose through upregulation of glucose transporters. Furthermore, the increased production of lactate will stimulate metastasis due to the degradation of extracellular matrix, block mitochondria-initiated apoptosis and stimulate angiogenesis through activation of the VEGF/R pathway [48–50]. The main drive behind aerobic glycolysis is thought to be hypoxia, which activates hypoxia-inducible factor 1α (HIF-1α), followed by upregulation of GLUT1, pyruvate dehydrogenase kinase 1 (PDK1) and LDHA [51].

## Altered metabolism in MM

### Glucose metabolism

**Fig. 2:**
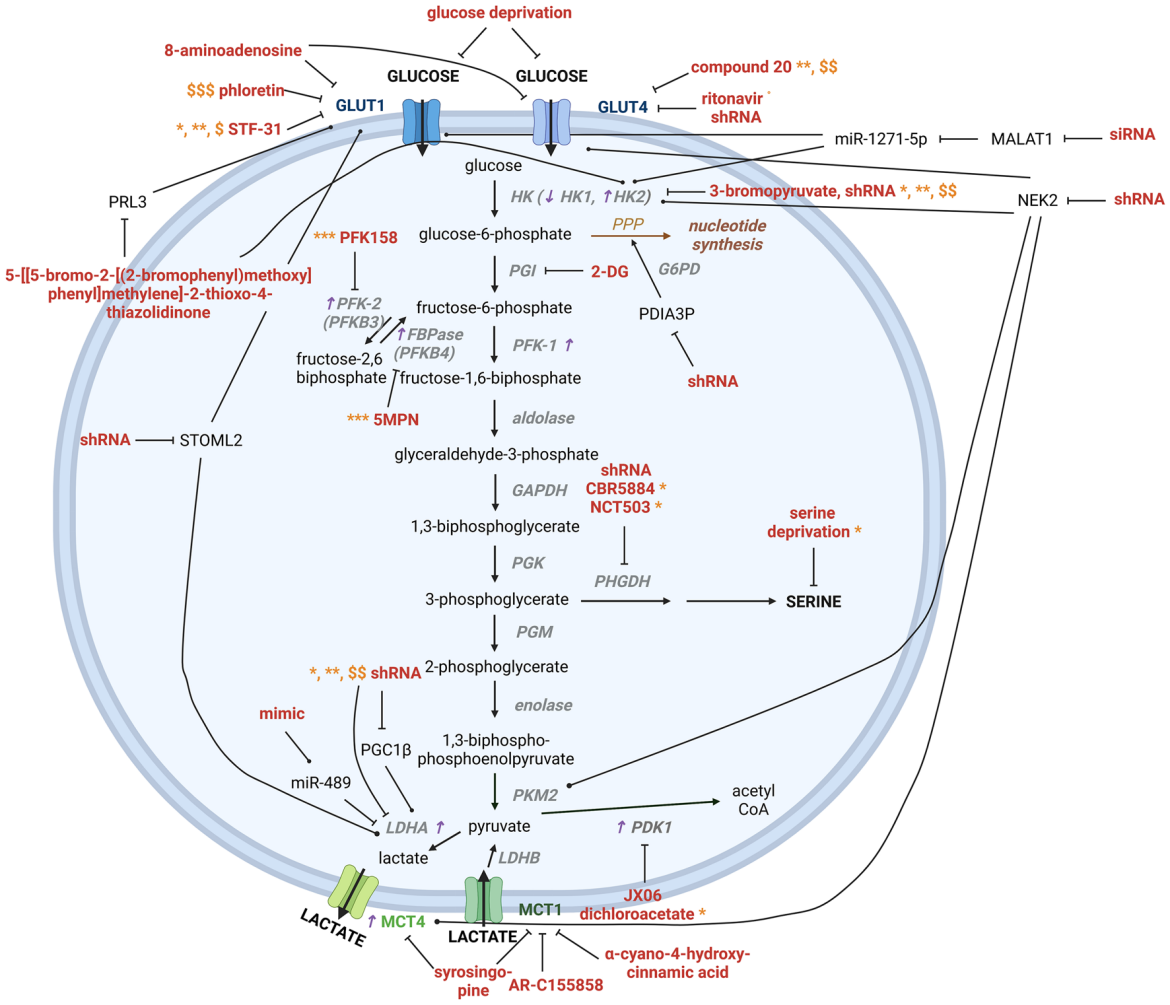
Overview of the altered glucose and lactate metabolism identified in myeloma. Glucose and lactate metabolism have been targeted in MM at several stages during the metabolic process; uptake, enzymatic conversion and export. Glucose transport in myeloma was blocked by targeting GLUT transporters (8-aminoadenosine, phloretin, STF-31, compound 20, ritonavir and siRNA/shRNA) or limiting the amount of glucose present for uptake (deprivation). Apart from the uptake, several glycolytic enzymes have also been targeted to reduce glycolytic function by inhibitors (3-bromopyruvate, 2-DG, 5MPN, PFK158 and JX06, dichloroacetate) and/or shRNAs. Moreover, serine metabolism can be inhibited by affecting PHGDH through shRNA, CBR5884 and NCT503. Serine deprivation also reduced MM viability. Lactate import through MCT1 was blocked by several metabolic inhibitors: AR-C155858, α-cyano-4-hydroxy-cinnamic acid and syrosingopine, while lactate production and export has been targeted through shRNA and syrosingopine. ACSS2 targeting by shRNA inhibited acetyl-CoA production. Used inhibitors, genetic approaches (siRNA/shRNA) and deprivations to affect these metabolic pathways are shown in red. Purple arrows indicate increase or decrease in a hypoxic environment. Enzymes are shown in grey. Successful combination approaches, resulting in lower viability or higher apoptotic cell death are shown in orange: *bortezomib, **melphalan, ***carfilzomib, $doxorubicin, $$dexamethasone, $$$daunorubicin, °metformin, °°venetoclax, °°°MG-123. *HK: *hexokinase, *PGI:* phosphoglucose isomerase, *PFK:* phosphofructokinase, *GAPDH:*  glyceraldehyde-3-phosphate dehydrogenase, *PGK:* phosphoglycerate kinase, *PGM:* phosphoglyceromutase, *PK: *pyruvate kinase, *LDH:* lactate dehydrogenase, *PDK: *pyruvate dehydrogenase kinase, *MCT: *monocarboxylate transporter, *GLUT: *glucose transporter, *shRNA: *short hairpin ribonucleic acid, *siRNA: *small interfering ribonucleic acid, *PPP: *pentose phosphate pathway, *G6PD: *glucose-6-phosphate dehydrogenase, *FBPase:* fructose-1,6-biphosphatase, *PRL-3: *phosphatase of regenerating liver 3, *STOML2: *stomatin-like protein 2, *5MPN: *5-(*n*-(8-methoxy-4-quinolyl)amino)pentyl nitrate, *STF-31: *4-[[[[4-(1,1-dimethylethyl)phenyl]sulfonyl]amino]methyl]-*N*-3-pyridinyl-benzamide, *PGC1β:* peroxisome proliferator-activated receptor-gamma (PPAR-gamma) coactivator-1 beta, *PDIA3P: *protein disulfide isomerase family A member 3 pseudogene 1, *2-DG: *2-deoxyglucose, *MALAT1: *metastasis associated lung adenocarcinoma transcript 1, *NEK2:* NIMA-related kinase 2, *ACSS2:* acyl CoA synthetase short. Created with BioRender.com

MM cells heavily depend on glycolysis, as shown by their increased glycolytic gene profile and their sensitivity to glycolysis inhibitors [52, 53]. First, glucose uptake has been targeted in MM through numerous ways (Fig. 2). GLUT1 inhibition by STF-31 induced apoptosis in several MM cell lines as a single agent, but also increased melphalan, doxorubicin and bortezomib-mediated cell death [54]. GLUT4 inhibition by short hairpin ribonucleic acid (shRNA) and ritonavir reduced MM cell viability in multiple MM cell lines and in primary patient samples [55, 56]. However, some MM cells were able to survive the lack of glucose/ritonavir treatment by changing their preferred metabolism to glutaminolysis. Additionally, treating MM cells with ritonavir also increased their sensitivity to metformin, an anti-diabetic drug that targets mitochondrial complex 1 involved in oxidative phosphorylation. Moreover, this combination strategy induced apoptosis in multiple MM cell lines and patient samples, and more importantly reduced tumor burden in vivo [57]. GLUT4 inhibition by compound 20 also resensitized MM cells to melphalan and dexamethasone treatment [56]. Another research group used 8-aminoadenosine, a purine analogue, to reduce GLUT expression and limit the uptake of glucose into MM cells. They observed reduced viability in several MM cell lines and reduced intracellular ATP levels. After 24 h of treatment, both GLUT1 and GLUT4 expression were decreased in the myeloma cell line MM.1S. Additionally, treatment with 8-aminoadenosine decreased glucose consumption and activated autophagy, but no cell death was observed in the MM.1S cells. By contrast, the more drug-resistant U266 cell line showed apoptotic cell death, but minor-to-no changes in GLUT1 and GLUT4 expression. The researchers explained this difference by the fact that the U266 cells are a more resistant cell line, with a deficiency in autophagy, which is not able to alter its metabolism in the absence of glucose, thereby triggering cell death [58].

Another way to target glucose uptake is through the glucose analogue 2-deoxyglucose (2-DG) (Fig. 2). After uptake into the cell, 2-DG is phosphorylated into 2-deoxyglucose-6-phosphate by hexokinase 2 (HK2). No further metabolization is possible, causing an accumulation of 2-DG in MM cells, affecting regular glycolysis [23, 59, 60]. Schibler et al. confirmed that 2-DG induces endoplasmatic reticulum stress in multiple MM cell lines, and that when combined with the mitochondrial-targeting agent decyl-triphenylphosphonium (10-TPP) it induced apoptosis in MM [61]. Glucose uptake can also be reduced by treating MM cells with the phosphatase of regenerating liver 3 (PRL-3) inhibitor 5-[[5-Bromo-2-[(2-bromophenyl)methoxy]phenyl]methylene]-2-thioxo-4-thiazolidinone which leads to dose-dependent apoptotic cell death [62, 63]. Moreover, shRNA against stomatin-like protein 2 (STOML2), a protein involved in the biogenesis and activity of mitochondria, also reduced glucose uptake, LDHA and HK2, leading to reduced proliferation of MM cells [64].

Second, apart from the glucose uptake, several glycolytic enzymes have also been targeted in MM to reduce myeloma progression (Fig. 2). HK2 was targeted in MM by the small molecule inhibitor 3-bromopyruvate [65]. Niedzwiecka et al. showed that 3-bromopyruvate reduces glutathione concentrations in RPMI-8226 MM cells after 2 h of treatment. Moreover, the amount of viable cells was decreased, while the percentage of necrotic cells increased. A slight increase in late apoptotic cells was also observed [65]. Moreover, Caillot et al. further confirmed the importance of HK2 in MM as they showed that HK2 expression is increased in MM cells compared to normal plasma cells, MGUS and SMM cells and that HK2 expression is associated with a poor prognosis in myeloma [66].

Further downstream, PKM2 is involved in the conversion of 1,3 biphospho-phosphoenolpyruvate into pyruvate. c-MYC regulates PKM2 expression through the NEK2/hnRNPA1/2 complex. This complex binds exon 9 of PKM pre-mRNA, cutting exon 9 out, leading to increased expression of PKM2 and increased glycolysis. Knockdown of NEK2 by shRNA decreased expression of several glycolysis-related genes, including GLUT4, HK2, LDHA and MCT4 (Fig. 2). Also, MM patients with high NEK2 and PKM2 expression show lower overall and event-free survival compared to patients with low expression of these genes [57].

A recent study highlighted the metabolic role of PDK1, which inactivates pyruvate dehydrogenase in the mitochondria, changing its preferred metabolism to aerobic glycolysis instead of oxidative phosphorylation. The novel PDK1 inhibitor JX06 successfully reduced MM cell growth and stimulated apoptosis, while limiting glucose metabolism [67] (Fig. 2). Similarly, PDK1 targeting by dichloroacetate also reduced proliferation and increased apoptosis, both as a single agent and in combination with bortezomib. These results were confirmed in vivo, where combination therapy significantly prolonged survival of myeloma-bearing mice [68].

PGC1β has been shown to increase LDHA expression, thereby promoting glycolysis, proliferation and MM cell growth. Knockdown of either PGC1β or LDHA inhibited glycolysis, leading to an increased generation of Reactive Oxygen Species (ROS) and apoptosis. In vivo, their inhibition prolonged survival [69] (Fig. 2). Fuijwara et al. further confirmed these results by showing that high LDHA expression correlates with a significant lower overall survival in MM patients [68]. Another research group identified microRNA miR-489 as a stimulator of aerobic glycolysis. MicroRNAs are defined as small non-coding RNAs, 18–25 of nucleotides in length and primarily interact with the 3’ UTR of mRNAs to post-transcriptionally inhibit their expression [70, 71]. In the last decade, microRNAs have become of great significance in cancer research. In MM, miR-489 expression was found to be downregulated. Gain-of-function experiments to overexpress miR-489 decreased MM cell viability, proliferation and aerobic glycolysis by targeting LDHA [72].

Comparing serum from MM patients to healthy controls also identified the long non-coding RNA (lncRNA) MALAT1 as being able to alter glucose and lactate metabolism in MM. LncRNAs are identified as transcripts consisting of over 200 nucleotides in length, but not translated into proteins. They primarily function as regulators of gene expression [73, 74]. LncRNA MALAT-1 was found to be significantly increased, while miR-1271-5p was significantly decreased in MM serum. SiRNA-mediated knockdown of MALAT-1 reduced glucose consumption and lactate production, and expression of glycolysis-related genes HK2 and GLUT1 were also decreased on protein level (Fig. 2). MiR-1271-5p inhibition abolished the previously seen effects of MALAT1 knockdown on the glycolytic pathway in MM [75]. Another lncRNA, protein disulfide isomerase family A member 3 pseudogene (PDIA3P), is also involved in MM drug resistance, as it affects glucose-6-phosphate dehydrogenase (G6PD) and the pentose phosphate pathway (PPP). PDIA3P binds the G6PD promotor through c-MYC, increasing G6PD expression and PPP flux. Short hairpin mediated knockdown of PDIA3P significantly reduced proliferation in U266 cells, both as a single agent and in combination with bortezomib [76].

### Lactate metabolism

Lactate transport via the MCT importers and exporters is also implicated in MM. Cancer cells show an increased activation of glycolysis to overcome their high need for energy, followed by an increased lactate production. This increase in lactate is accompanied by an important decrease in pH. To avoid cellular acidosis, cancer cells must adapt by increasing the proton efflux through upregulation of MCTs among others. MM cells have been shown to overexpress MCT1 and MCT4. MCT4 interference did not affect MM proliferation in human MM cell lines, while silencing of MCT1 non-significantly decreased lactate export and increased cellular pH [77] (Fig. 2). MM cell lines treated with the MCT1 inhibitor α-cyano-4-hydroxycinnamic acid also showed a decrease in lactate uptake and increase in apoptotic cell death [77, 78]. Hanson et al. investigated MCT1 and MCT2 in MM, and observed that MCT1/MCT2 inhibition caused a decrease in lactate export, followed by a decrease in intracellular pH, ultimately leading to cell death [79]. Benjamin et al. showed that metformin and its more potent analogue phenformin, resensitize MM cells (amongst other cancer cell types) to syrosingopine-mediated MCT1/MCT4 targeting, leading to apoptotic cell death in the MM cell line OPM-2 [80].

### Amino acid metabolism

A large proteome profiling study from 2019 on 10 MM patients, one MGUS patient and two SMM patients showed alterations in the glutamine pathway. Here they found that MM cells derived from patients with over 40% of BM infiltration showed a high glutamine uptake as indicated by the high expression of the glutamine transporter SLC1A5 [81]. Pyrroline-5-carboxylate reductase (PYCR) enzymes were also upregulated, which convert glutamine into proline. Moreover, proline and arginine metabolism were found to be altered in both the plasma and BM of MM patients [81].

**Fig. 3:**
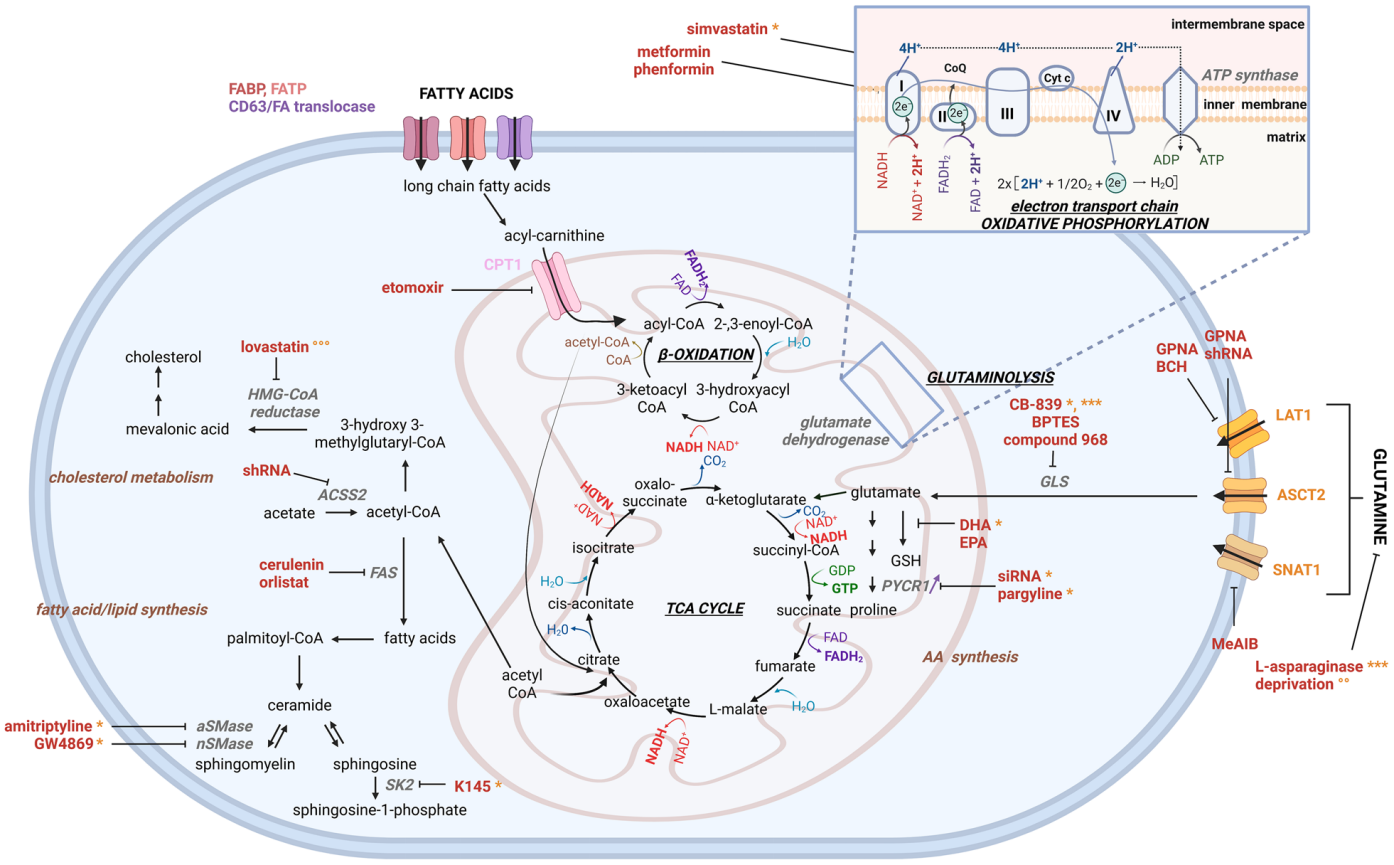
Overview of the altered glutamine and fatty acid metabolism identified in myeloma. Glutamine and fatty acid metabolism have been targeted in MM at several stages during the metabolic process; uptake and enzymatic conversion. Glutamine metabolism can be targeted by either affecting the glutamine uptake (by inhibitors GPNA, BCH, MeAIB or shRNA) or depriving the cells of glutamine by deprivation or l-asparaginase. Conversion of glutamine to glutamate is blocked by CB-839, BPTES and compound 968, while conversion to other metabolites was blocked by DHA and EPA for GSH production, as well as pargyline and siRNA for proline production. Uptake of acyl-carnithine into the mitochondria through CPT1 has been blocked in MM by etomoxir, lovastatin, cerulenin and orlistat affect acetyl-CoA metabolism. Amitriptyline, GW4869 and K145 affect sphingolipid metabolism, while simvastatin, metformin and phenformin affect complex I and II of the electron transport chain. Used inhibitors, genetic approaches (siRNA/shRNA) and deprivations are shown in red. Enzymes are shown in grey. Purple arrows indicate increase or decrease in a hypoxic environment. Successful combination approaches are shown in orange: *bortezomib, **melphalan, ***carfilzomib, $doxorubicin, $$dexamethasone, $$$daunorubicin, °metformin, °°venetoclax, °°°MG-123. *CPT1: *carnithine palmitoyltransferase I, *GSH: *glutathione, *GPNA: l*-γ-Glutamyl-*p*-nitroanilide, *BCH:* 2-aminobicyclo-(2,2,1)-heptane-2-carboxylic acid, *MeAIB: *α-(Methylamino)isobutyric acid, *BPTES: *Bis-2-(5-phenylacetamido-1,3,4-thiadiazol-2-yl)ethyl sulphide, *DHA: *docosahexaenoic acid, *EPA: *eicosapentaenoic acid, *siRNA: *small interfering ribonucleic acid, *AA: *amino acid, *FAS: *fatty acid synthase, *HMG: *β-hydroxy β-methylglutaryl, *SK-2: *sphingokinase 2, *aSMase: *acid sphingomyelinase, *nSMase: *neutral sphingomyelinase, *LAT1:* large amino acid transporter 1, *ASCT2:* alanine, serine, cysteine transporter 1, *SNAT1:* sodium-coupled neutral acid transporter 1, *GLS:* glutaminase, *PYCR:* pyrroline-5-carboxylate reductase, *GSH: *glutathione. Created with BioRender.com

Bolzoni et al. have shown that MM cells are glutamine-dependent, and that primary CD138 + MM cells show an overexpression of glutamine transporters SNAT1, ASCT2 and LAT1 [82]. Although glutamine is a non-essential amino acid, the authors have shown that MM cells depend on the uptake of extracellular glutamine, and that uptake inhibition by ASCT2 downregulation inhibited cell growth in several human MM cell lines (Fig. 3). The same group also tested several amino acid analogues (MeAIB, GPNA and BCH), and found that GPNA, which blocks glutamine uptake through the ASCT2 transporter, was able to significantly reduce glutamine uptake by 60% [82] (Fig. 3). Moreover, GPNA and BCH (targeting glutamine uptake through LAT1) were able to reduce cell viability, while MeAIB (SNAT1) showed only a small effect on viability. Specificity of GPNA was confirmed through shRNA-mediated knockdown. In vivo, mice injected with JJN3 cells with knockdown for ASCT2 showed significant lower tumor volumes compared to the scrambled control mice [82].

MM cells also undergo apoptosis when cultured in the absence of glutamine or in the presence of l-asparaginase. This metabolic drug is FDA-approved for the treatment of acute lymphoblastic leukemia (ALL) and hydrolases asparagine but also glutamine. Combined with carfilzomib, l-asparaginase increases ROS-mediated cell death [83, 84]. Bajpai et al. showed that MM cells surviving glutamine deprivation, will upregulate BIM expression and promote BIM’s binding to BCL-2, rendering the MM cells more sensitive to venetoclax. Moreover, they also showed that this gained venetoclax sensitivity can be reversed by supplementing the cells with α-ketoglutarate, an intermediate in the Krebs cycle. Their research confirms the adaptability of MM cells to change their preferred metabolism to avoid cell death. However, these induced metabolic changes can again be exploited by researchers to achieve synthetic lethality [85].

Inhibition of glutaminase, the enzyme that converts glutamine into glutamate, by several small molecule inhibitors (compound 968, BPTES) successfully induced apoptotic cell death and reduced viability in numerous MM cell lines, except for the more resistant U266 cells (Fig. 3) [82, 87]. This apoptosis was mediated by MYC degradation, which U266 cells lack. Importantly, the decrease in c-MYC levels in the INA6 cell line was already visible 20 min after glutamine was deprived [87].

The fact that MM cells heavily depend on glutamine uptake and metabolism is further confirmed by the use of [^18^F](2S,4R)-4-fluoroglutamine as a tracer for Positron Emission Tomography in Myeloma in preclinical models. Voltarta et al. showed high uptake of [^18^F](2S,4R)-4-fluoroglutamine in two murine MM models. Tracer uptake was significantly reduced after bortezomib treatment. Importantly, mice with highest bortezomib response showed an even further decline in fluoroglutamine uptake, but not in fluorodeoxyglucose uptake [86]. Moreover, other amino acid-based or amino acid-derivatives radiopharmaceuticals, e.g. [^11^C]-methionine, [^18^F]-fluorethlytyrosine and [^11^C/^18^F]-choline have also successfully been tested in preclinical MM models and/or MM patients [88, 89].

Finally, a gut microbiome study in MM revealed that *Klebsiella pneumoniae* stimulates the progression of MM by promoting the de novo synthesis of glutamine in 5TGM1 mice. Furthermore, the absence of glutamine in their diet reduced MM progression in vivo, as indicated by the reduction in tumor fluorescence intensity [90].

### Lipid metabolism

A profiling study from 2018 identified large differences in amino acid, lipid and energy metabolism profiles comparing NDMM, RRMM, MGUS and healthy controls [91]. Significant changes were found between the different groups including free carnithine, four species of phosphatidylcholine (PC), acetylcarnithine, asymmetric dimethylarginine (ADMA) and glutamate when comparing NDMM to MGUS, while octadecanoylcarnithine, ADMA and six species of PCs were altered comparing RRMM to MGUS. When comparing RRMM to NDMM, the concentrations of free carnithine, creatinine, acetylcarnitine, five lyso PCs and PCs were significantly altered. These data confirm that not only does the metabolic profile from MM patients differ from healthy controls, this also changes during MM progression [92]. Another large metabolomics study compared the BM supernatants and plasma from healthy volunteers and MM patients in ISS stage I–III. They observed a distinct metabolic shift depending on the ISS stage. They found changes in fatty acid metabolism, with a decrease in glycerol, palmitic acid, oleic acid and linoleic acid in BM of MM patients compared to healthy controls. The authors hypothesized that this decrease in fatty acids might be due to an increased use of fatty acids for membrane biosynthesis necessary for the proliferation of clonal plasma cells [93].

Our research group has also investigated changes in lipid metabolism in MM [94]. We performed a large lipidomics study, identifying the dysregulation of several lipids when comparing the plasma of MM patients to healthy controls. Top upregulated lipids were several ceramides and phosphatidylethanolamines, while several PC, sphingomyelin and one species of phospatidylethanolamine were downregulated. The imbalance between sphingomyelin and ceramide suggested an increased activity or expression of the enzyme sphingomyelinase, which we confirmed in primary patient samples. Inhibiting acid sphingomyelinase by amitriptyline or neutral sphingomyelinase by GW4869 significantly increased bortezomib and melphalan-mediated cell death in several cell lines [94] (Fig. 3). Moreover, another study from 2021 identified that MM cells show an accumulation of lipids upon treatment with the proteasome inhibitor ixazomib. Lipid inhibitor lovastatin enhanced the apoptotic effects of the proteasome inhibitor MG-132 in two MM cell lines; MM.1S and RPMI-8226 [95]. Chen et al. observed that pre-treatment of MM cells with docosahexaenoic acid (DHA) or eicosapentaenoic acid (EHA) increased bortezomib sensitivity by lowering glutathione levels and altering metabolites and enzymes in the glutathione metabolic pathway (Fig. 3). Simultaneous treatment (bortezomib + DHA or bortezomib + EHA) however caused only minor changes in MM [29].

Jurczyszyn et al. investigated the differences in fatty acid composition of red blood cell membranes between MM patients and healthy controls [96]. They observed that red blood cells of MM patients contain higher levels of saturated fatty acids and n-6 polyunsaturated fatty acids (PUFA), while the presence of monounsaturated, n-3 PUFA and trans fatty acids was lower in the red blood cell membranes [96]. Moreover, the same research group showed in a later paper that the amount of saturated fatty acids and n-6 PUFA was also increased in the plasma of MM patients, suggesting an increase in fatty acid synthesis in MM [97]. Moreover, it has been shown that MM cell lines have an increase in fatty acid oxidation in response to decreased glycolysis, but also an increase in fatty acid synthase expression [98–100]. Both the inhibition of fatty acid oxidation by etomoxir and fatty acid synthesis by orlistat reduced in vitro proliferation, whereby combination therapy even increased these effects [99, 100]. Wang et al. also investigated fatty acid synthase in MM, and observed an important increase in fatty acid synthase levels in 70% of tested MM patients, while it was undetectable in matched healthy volunteers. Moreover, inhibition of fatty acid synthase by cerulenin induced apoptosis in MM cell lines [101]. Li et al. also investigated acetyl-CoA synthetase 2 (ACSS2), which converts acetate to acetyl-CoA, an important metabolite for the TCA cycle. ACSS2 expression is increased in MM cells from obese patients, and inhibition of this enzyme by shRNA reduced MM cell growth both in vitro and in vivo (Fig. 3). Finally, increased ASCC2 expression in MM cells was attributed to stimulation of the adipose tissue [102].

### Other metabolic alterations

Aside from the genetic alterations that greatly impact myeloma development, epigenetic aberrations, including changes in DNA and histone methylation contribute to myeloma progression. EZH2 is a methyl transferase that catalyzes H3K27me3 methylation. EZH2 inhibition by UNC1999 in MM significantly reduced tumor load in vivo and viability in vitro in several human MM cell lines. Moreover, UNC1999 stimulated betaine and methionine metabolism in INA-6 cell line, which is sensitive to EZH2 inhibition. UNC1999-resistant cell lines also showed non-significant upregulation in their betaine and methionine metabolism alongside a significant increase in their homocysteine degradation. Metabolic profiling revealed increases in 5-methyltetrahydrofolic acid, 5′ methylthioadenosine and homocysteine and a decrease in glycine in INA-6, while no significant changes in these metabolites were found in the UNC1999-resistant U1996 cells. Other metabolic genes, including methionine adenosyltransferase 2A, methionine adenosyltransferase 2B, cystathionine-beta-synthase and cystathionase were also decreased when cells were sensitive to UNC1999 [103].

### Drug resistance-related metabolic alterations in MM

Several metabolic alterations related to drug resistance have been investigated in MM, mostly by comparing drug sensitive and resistant cell lines. Bortezomib resistance in MM correlates with increased mitochondrial function, as evidenced by increased expression of several mitochondrial genes like cyclophilin D, superoxide dismutase 2 and mitochondrial calcium uniporter [104]. Moreover, expression of several oxidative phosphorylation-related genes was found increased in patients resistant to bortezomib [105]. This increase in mitochondrial function was glutamine-driven. Moreover, bortezomib-resistant cells showed higher oxygen consumption rates, while bortezomib-sensitive and -resistant cells showed similar levels of extracellular acidification rates (ECAR), indicating that bortezomib resistance affects oxidative phosphorylation. Inhibition of glutaminase I by CB-839 increased sensitivity to bortezomib and carfilzomib in resistant MM cell lines [105] (Fig. 3). Tibullo et al. reported that bortezomib-resistant U266 cells contain a significant higher amount of mitochondria, GTP, UTP and CTP levels, while their glycolysation is also increased compared to bortezomib-sensitive cells [106].

Aside from higher oxidative phosphorylation rates, bortezomib-resistant MM cells also show increased glycolytic rates. Although Thompson et al. did not observe a difference in ECAR rates upon bortezomib resistance [105], Maiso et al. found that higher glycolytic activity caused drug resistance to bortezomib in a hypoxic environment. Moreover, LDHA inhibition combined with bortezomib increased apoptotic cell death compared to both single agents [107] (Fig. 2). The group of Berkers found that changes in the serine biosynthesis from glucose were associated with drug resistance, whereby phosphoglycerate dehydrogenase (PHGDH) is upregulated in bortezomib-resistant cells. They also confirmed a higher glucose uptake in the resistant cells. When these MM cells were deprived of serine, their sensitivity to bortezomib increased, overcoming the resistance [108] (Fig. 2). Elsaadi et al. [109] treated several proteasome sensitive and resistant MM cell lines with two different PHGDH inhibitors: CBR5884 and NCT-503. Both inhibitors reduced MM viability, and increased bortezomib-mediated effects in almost all MM cell lines. Observed effects were confirmed using PHGDH knockdown through shRNA in MM cell lines, while combination therapy of NCT-503 and bortezomib reduced tumor load in vivo with 35% compared to bortezomib alone [109] (Fig. 2). Wu et al. [110] observed similar effects, showing that PHGDH overexpression stimulates MM cell growth and bortezomib resistance and NCT-503 also potentiated bortezomib-mediated effects on cell growth (Fig. 2). When investigating the pathway of action, they found that PHGDH increased glutathione synthesis, limiting ROS generation and stimulating cell growth [110].

Another potential metabolic target in MM is the mevalonate pathway, which is responsible for synthesis of multiple lipids including cholesterol, ubiquinone and dolichol. Bortezomib-resistant MM cells show increased activity of both the TCA cycle and oxidative phosphorylation, alongside high presence of the mitochondrial electron carrier CoQ. CoQ is a crucial factor in the activity of electron transport chain complex I and complex II, but is also produced during the mevalonate pathway. Inhibition of CoQ through simvastatin reduced cell viability in bortezomib-resistant MM cells, but had only small effects on wild type MM cells [111] (Fig. 3).

Lipid metabolism is also altered in drug-resistant MM cells. Sphingosine kinase 2 is responsible for the phosphorylation of sphingosine, producing sphingosine-1-phosphate. The sphingosine kinase 2 inhibitor K145 significantly increased bortezomib-mediated cell death in both bortezomib-sensitive and -resistant cell lines by upregulating the unfolded protein response pathway [112, 113] (Fig. 3).

## Role of the tumor microenvironment in MM metabolism

The BM plays an important role in the metabolic features of MM cells and the surrounding cell types, which was recently confirmed by Fei et al. [93]. They performed a large metabolomics study on plasma and BM samples, comparing healthy individuals to MM patients. Vulcano plots identified different metabolic changes, comparing plasma to BM. In plasma samples of MM patients, six metabolites were increased compared to plasma of healthy volunteers: urea, uric acid, proline, xanthine, aspartate and creatinine, while the amount of serine was decreased. For BM samples, threonine, glutamine, glycerol, valine, linoleic acid, oleic acid, histidine, palmitic acid and 3-hydroxybutyric acid were decreased in MM patients, while glutamate, aspartate, urea, malate, succinate, ornithine and xanthine were increased. Comparing BM to plasma, only urea, aspartate, creatinine and xanthine were upregulated in both samples. Enrichment analysis showed that arginine metabolism, proline metabolism and the urea cycle are disturbed in both BM and plasma of MM patients, while changes in glutamate metabolism, the TCA cycle and carnitine synthesis were only found in the BM [93].

### Hypoxic niche

As mentioned earlier, the hypoxic nature of the natural BM microenvironment leads to the universal Warburg effect, whereby cancer cells change their preferred metabolism to aerobic glycolysis with high production of lactate [46, 47]. Hypoxia-inducible factor (HIF) is a transcription factor that regulates expression of several genes involved in the response to hypoxia. HIF consists out of five subunits: 3 α-subunits and 2 β-subunits: HIF-1α, HIF-2α, HIF-3α, HIF-1β and HIF-2β. In normoxic conditions, HIF-1α is hydroxylated at specific proline residues and then recognized by von Hippel-Lindau E3 ubiquitin ligase (VHL) and subsequently degraded through the proteasome. When oxygen levels fall below 5%, HIF-1α translocates to the nucleus, where it will form a dimer with HIF-1β and become a stable complex, protected from degradation through the proteasome. The dimer is then able to bind to hypoxia response element (HRE) and influence expression of target genes [114, 115]. HIF-1α activation triggers HIF-2α activation and functions as a central controller to adapt to chronic hypoxia through increased expression of its targets [116, 117]. Both HIF-1α and HIF-2α play important roles in MM progression. HIF-1α activation promotes glycolysis through upregulation of several glycolysis-related genes, including transporters, hexokinase and pyruvate dehydrogenase kinase [118, 119]. At the same time, the mitochondrial oxidative phosphorylation is reduced. The significance of HIF-3α has not yet been confirmed [116].

The same profiling study from 2019 investigating the metabolome of both BM and plasma found that several hypoxia-related genes involved in mitochondrial translation were dysregulated in MM cells. They observed a reduced presence of HIF1AN and VHL, both proteins which are involved in the prevention of HIF activation. PDPK1, a key enzyme in hypoxia-responsive metabolism change, and TNF receptor associated protein 1 (TRAP1), a mitochondrial chaperone, were also increased in MM cells with > 40% of BM infiltration [81]. Hexokinase 1 was decreased, pointing to a reduced glycolysis in advanced MM [81]. Downstream targets of the hypoxia-induced miR-210, including TP53I11 and PTPN1, were also decreased. Remarkably, hypoxia-regulated Fyn was highly expressed in MM with low infiltration, while it showed lower expression in MM with high infiltration [81].

**Fig. 4:**
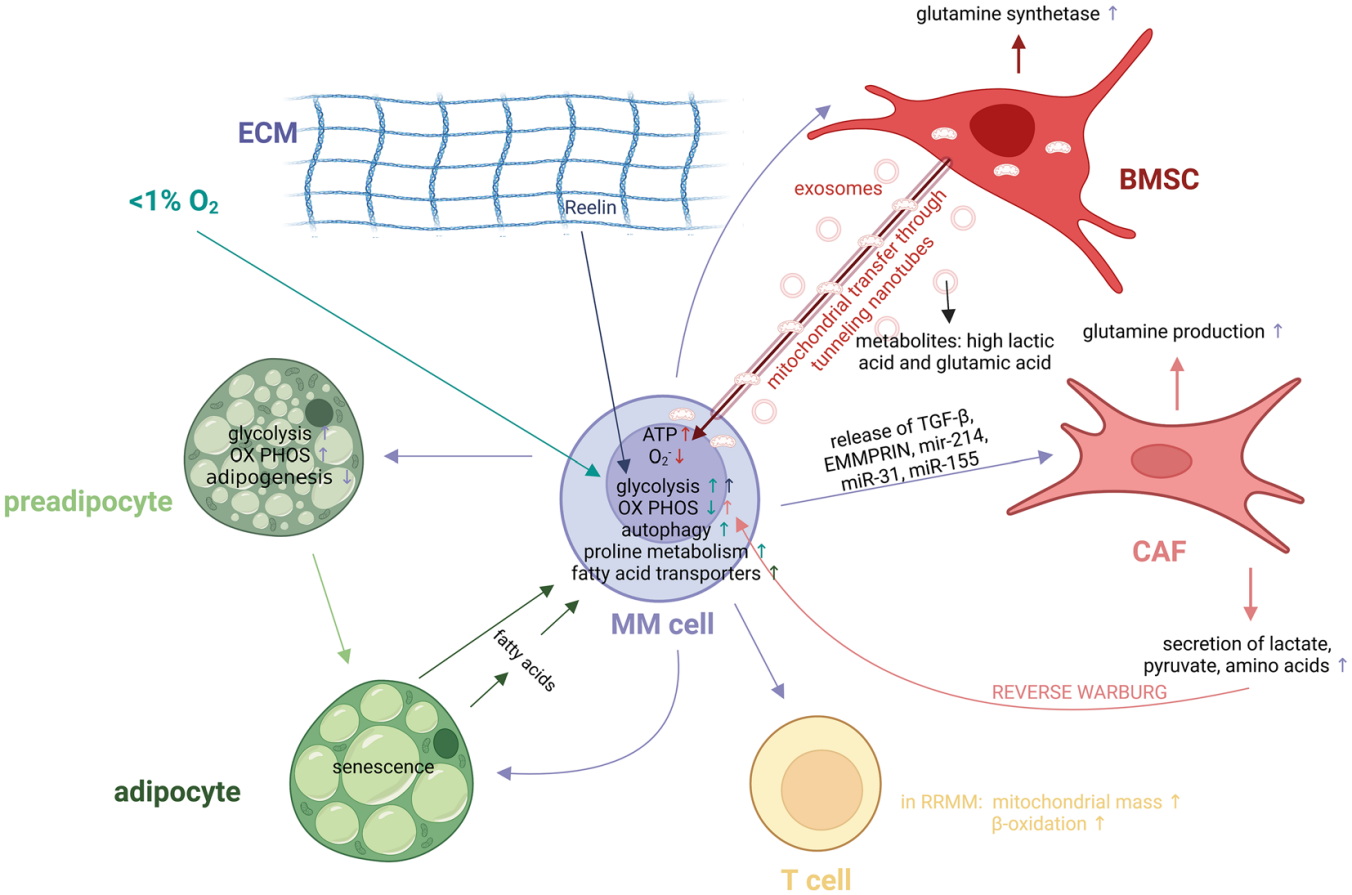
Overview of the metabolic interactions between MM cells and other components of the BM environment. MM cells are able to influence metabolism of neighboring cell types in the BM microenvironment and vice-versa. BMSC transfer their mitochondria to MM cells through tunnelling nanotubes, increasing ATP values and decreasing superoxide concentrations. Moreover, BMSC-derived exosomes contain high levels of lactic acid and glutamic acid, which is hypothesized to affect MM cell metabolism. MM cells also stimulate stromal cells to increase their glutamine production by upregulation of glutamine synthetase. MM cells release several factors, including TGF-β, EMMPRIN and miR-214, miR-31 and miR-155, stimulating CAFs to increase their glutamine production and secretion of lactate, pyruvate and other amino acids. Known as the Reverse Warburg effect, these metabolites are then taken up again by the MM cells, stimulating oxidative phosphorylation in the MM cells. T-lymphocytes of RRMM patients also show metabolic alterations, including an increase in mitochondrial mass and β-oxidation. Preadipocytes show an increase in glycolysis, oxidative phosphorylation and decrease in adipogenesis, induced by the neighboring MM cells. Moreover, MM cells stimulate adipocytes to produce and release fatty acids, which in turn is used by the MM cells as nutrients. MM cells will also upregulate their fatty acid transporters. The hypoxic BM environment greatly influences MM metabolism by upregulating glycolysis, autophagy and proline metabolism. Reduced levels of oxidative phosphorylation were also observed. Finally, ECM-protein Reelin increases glycolysis in MM cells. *ECM: *extracellular matrix, *BMSC:* bone marrow stromal cell, *CAF: *cancer-associated fibroblast, *OX PHOS:* oxidative phosphorylation, *MM:* multiple myeloma, *RRMM:* relapsed/refractory multiple myeloma, *BM: *bone marrow. Created with BioRender.com

In 2020, Ikeda et al. demonstrated that hypoxic stress can upregulate HK-2, an enzyme involved in glycolysis, in MM cells that catalyzes the conversion of glucose into glucose-6-phosphate [120] (Fig. 2). Furthermore, the hypoxic environment induced autophagy, which is used by cancer cells to avoid drug-mediated cell death. Knockdown of HK2 decreased glycolysis and autophagy; and induced apoptosis in MM cell lines under hypoxic conditions [120] (Fig. 4). A recent study from 2018 investigating gene expression in primary MM samples as well as several human MM cell lines revealed that chronic hypoxia was able to increase the activity of several glycolysis-related enzymes, including fructose-2,6-biphosphatase (FBPase) and 6-phosphofructo-2-kinase (PFK2) (together known as PFKFB) [121] (Fig. 4). Okabe et al. also investigated these enzymes during MM progression, and confirmed the hypoxia-induced upregulation of two PFKFB enzymes: PFKFB3 (high kinase activity) and PFKFB4 (high phosphatase activity). Inhibition of these enzymes by PFK158 and 5MPN, respectively inhibited proliferation and cell growth (Fig. 2). Moreover, these inhibitors increased sensitivity to carfilzomib [122]. As mentioned earlier, Maiso et al. also showed that expression of HK-2 and LDHA increased upon hypoxic culture, leading to bortezomib, melphalan and dexamethasone resistance in multiple MM cell lines. Moreover, inhibition of these metabolic genes resensitized the MM cells to these drugs [107]. Phloretin, a GLUT1 inhibitor, increased the cytotoxic effects of daunorubicin in MM when cells were treated in a hypoxic environment [123] (Fig. 2).

We have recently shown that proline metabolism is altered in MM in hypoxic conditions. MM cells cultured in < 1% O_2_ show an increased glutamine-to-proline conversion, as well as higher levels of intracellular proline (Figs. 3, 4). SiRNA-mediated targeting of the PYCR1 enzyme, responsible for glutamine-to-proline conversion, inhibited proline production, decreased MM viability, proliferation and increased bortezomib-mediated cell death. Moreover, the PYCR1 inhibitor pargyline combined with bortezomib significantly reduced tumor load in vivo, compared to both single agents [124].

MM metabolism can also be regulated by cytokines. Aass et al. [125] showed that the presence of IL-32 is at least partly regulated by cysteamine dioxygenase and HIF-1α. Upon hypoxic treatment, MM cells increase intracellular IL-32 levels, thereby stimulating oxidative phosphorylation. Knockout of IL-32 in MM cells increased the presence of lipids, pyruvate precursors and citrate [125].

Raimondi et al. found that hypoxia decreased miR-199a-5p expression in OPM-2, RPMI-8226 and NCI-H929 cells. Reinforced expression of miR-199a-5p by mimics inhibited HIF-1α and SIRT-1 expression, reduced MM cell migration and increased the adhesion of MM cells to BMSCs under hypoxic conditions. Forced expression of miR-199a-5p also reduced proliferation and increased apoptotic cell death in hypoxic MM cells. Moreover, miR-199a-5p upregulation impaired the hypoxia-mediated release of pro-angiogenic factors, resulting in reduced endothelial cell migration [126, 127].

Moreover, aside from the MM cells, the hypoxic environment also causes changes to the surrounding cell types, for instance the osteoclasts. The hypoxic environment stimulates interleukin-32 release by MM cells through extracellular vesicles, which promotes osteoclast differentiation from pre-osteoclasts, leading to bone resorption. Pratt et al. showed that hypoxia increased peptidyl arginine deiminase 2 (PADI2) in BMSCs, which changed their metabolism by upregulating several glycolytic enzymes [128].

As the hypoxic environment causes a considerable shift in metabolic preference, our research group aimed to target this phenomenon in MM. TH-302 is a prodrug that is only activated into bromo-isophosphoramide in the presence of hypoxia. In vitro, the drug induced a G0/G1 cell-cycle arrest and triggered apoptosis under hypoxic conditions. No significant changes were observed in normoxic conditions. In vivo, myeloma-bearing mice showed lower M-protein concentrations and reduced microvessel density when treated with TH-302 at three different concentrations [129]. A phase I/II clinical trial in MM where patients were treated with TH-302 + dexamethasone or TH-302 + bortezomib + dexamethasone showed that TH-302 was well tolerated with or without bortezomib, but failed to significantly prolong survival [130].

### Interaction with stroma

Recently, the effect of mitochondrial transfer between MM and stromal cells has been investigated. Matula et al. showed that primary MM cells are able to resist cell death induced by cytotoxic agents by uptake of stromal cell-derived mitochondria [131]. After mitochondrial transfer, MM cells contain increased levels of ATP, while their mitochondrial superoxide levels decrease. Further investigation revealed that this mitochondrial transfer takes place through tunneling nanotubes and partial cell fusion. Remarkably, higher doses of cytotoxic agents also increased mitochondrial transfer (Fig. 4). Combination of cytotoxic agents with metformin blocked the supportive effect induced by stromal cells. Marlein et al. showed that mitochondrial transfer between stroma and MM cells is CD38-dependent [132]. They further observed that primary patient samples contain a higher basal oxygen consumption rate than MM cell lines. However, when NOD/SCID gamma mice were inoculated with MM cell lines, and then later isolated and tested, the MM cells growing in vivo also had higher oxygen consumption rates compared to control MM cells that grow in vitro. When primary BMSC are co-cultured with MM cell lines, the oxygen consumption rate was also increased in the MM cells (Fig. 4). This furthers supports the idea that cells growing in a microenvironment use more oxidative phosphorylation compared to monocells in vitro culture [132, 133].

Cancer-associated fibroblasts (CAFs) are a subtype of tumor stromal cells, able to metabolize glucose through aerobic glycolysis. This process is stimulated by the release of factors like TGF-β, EMMPRIN and microRNAs miR-214, miR-31 and miR-155 by cancer cells [134–137], causing the fibroblast to become activated CAFs and secrete metabolites including lactate, pyruvate and multiple amino acids [138–142] (Fig. 4). These nutrients are then taken back up by the surrounding cancer cells, which is referred to as the “Reverse Warburg effect”, stimulating oxidative phosphorylation and proliferation in the recipient cancer cells. Moreover, CAFs have also been shown to generate high levels of glutamine in solid tumors [143]. Our research group, in collaboration with the group of Vacca, has highlighted the role of CAFs in the myeloma BM microenvironment, where the MM cells are able to transform BMSCs into CAFs, which activate pro-survival pathways and induce drug resistance to bortezomib [144].

Importantly, MM cells are also able to influence stromal cells. It has been shown that the BM of MM patients contains lower concentrations of glutamine, induced by the high uptake and metabolism of glutamine inside the MM cells. This high uptake in MM cells also induces an increase in glutamine synthetase in neighboring mesenchymal stromal cells [132, 145, 146] (Fig. 4). Moreover, the glutamine-depleted BM environment inhibits mesenchymal stromal cell differentiation towards osteoblasts. Further investigation revealed that this inhibition can be restored by glutamine supplementation, and that both glutaminase and the glutamine transporter SNAT2 are required for osteoblast differentiation [146]. Moreover, asparagine is also an important amino acid for the asparagine synthetase-dependent generation of osteoblasts, and is generated from glutamine [147, 148].

The role of PKM2 in the adhesion between MM and BMSCs has been studied, as this process is crucial in the induction of drug resistance. Although we previously discussed PKM2 as a potential target for therapy, He et al. showed that PKM2 silencing by siRNA led to an increased adhesion of MM cells to BMSCs and induced cell adhesion-mediated drug resistance, as shown by lower bortezomib-induced apoptosis [149]. Therefore, PKM2 targeting in myeloma should be approached with care.

### Extracellular matrix

Aside from the cellular compartment, the non-cellular compartment can also influence metabolism in tumor cells. The extracellular matrix protein Reelin is involved in glycolysis in MM cells. Reelin knockdown in the MM cell line H929 reduced LDH, PDK1, lactate production and glucose uptake, while also reducing MM cell proliferation. Additional experiments revealed that Reelin contributes to MM cancer cell progression by increasing MM cell glycolysis through activation of the Syk/Akt and STAT3 pathways [150] (Fig. 4).

### Adipose tissue

BM adipocytes are another subtype present in the BM stroma. In MM, they form an important component of the tumor microenvironment. BM adipocytes contain triglycerides that produce and release fatty acids, which in turn can be taken up by surrounding MM cells [35, 150]. Moreover, another review article describing fatty acid metabolism in myeloma also hinted at a possible metabolic shift from aerobic glycolysis to fatty acid oxidation, as has been shown in leukemia. The authors further substantiate this metabolic change by mentioning that although MM cells heavily depend on glucose uptake, these cancer cells are mostly situated in a niche where high levels of adipocytes are present [35]. Fairfield et al. found that MM cells are able to inhibit adipogenesis and alter metabolic processes in preadipocytes [151, 152] (Fig. 4). MM patients contain significantly less BM adipocytes, a phenomenon that resets once patients are successfully treated. Upon coculture of MM cells and preadipocytes (3T3-L1), KEGG pathway analysis revealed an increase in oxidative phosphorylation and glycolysis in the preadipocytes before differentiation has started. These results were confirmed by gene set enrichment analysis, where a significant enrichment was found in genes involved in glycolysis and fatty acid metabolism among others (Fig. 4). In differentiating adipocytes, coculture with MM cells decreased lipid metabolism. Moreover, coculture initiated a senescent-phenotype in these adipocytes, confirming that MM cells are able to change their tumor microenvironment into a more favorable setting [151]. By contrast, adipocytes are also able to alter MM metabolism. MM cells stimulate adipocytes to break down lipids and release them to MM cells. Upon adipocyte signaling, MM cells upregulate fatty acid transporters on their cell membrane, stimulating MM cell proliferation. The authors concluded that blocking the uptake of free fatty acids may therefore be a novel treatment strategy in MM [153].

### Immune cells

Although not much is known yet about how metabolic changes in immune cells affect MM progression, Cooke et al. did compare the transcriptomic profile of T cells from NDMM to RRMM and found that two genes involved in fatty acid β-oxidation (acyl-CoA dehydrogenase, very long chain and acetyl-CoA acyltransferase 2) were increased in RRMM (Fig. 4). Moreover, T cells from RRMM showed higher mitochondrial mass, but reduced ROS and membrane potential, indicating a change in mitochondrial metabolism [154].

### Exosomes

Aside from cell–cell contact and secretion of soluble factors, the MM cells are also able to communicate with the BM microenvironment (and vice-versa) by exosomes, a subgroup of extracellular vesicles, 30–150 nm in diameter and released by endocytosis [155–160]. Their cargo mainly consists out of RNA, proteins, lipids and DNA [161]. Recipient cells will take up the exosomes by pinocytosis, endocytosis or plasma membrane fusion, after which their cargo will be released into the cytosol of the recipient cells [162]. Our research lab has investigated the role of exosomes in the development of myeloma and concluded that BMSC-derived exosomes induce drug resistance in MM tumor cells. Furthermore, MM tumor cell-derived exosomes contribute to niche-formation [160, 163] and interact with both osteoblasts and osteoclasts, stimulating bone resorption and inhibiting bone formation [164].

Until now, most researchers investigating the exosomal cargo have focused on proteins and RNAs. More recently, researchers have found that these exosomes also contain metabolites, which can be transferred to other cell types and influence the metabolism of these acceptor cancer cells, thereby stimulating their proliferation and survival [165, 166]. Importantly, cancer cells have been shown to release more extracellular vesicles compared to healthy cells [167, 168]. Although nothing is known yet about the metabolite cargo of MM exosomes, several researchers have already shown in other cancers and diseases that exosomes do contain metabolites. Puhka et al. performed metabolomics on extracellular vesicles (EVs) originating from urine and platelets from prostate cancer patients, confirming that EVs contain metabolites in their cargo. The most common metabolites found were subdivided into 5 main classes ordered by decreasing presence; organic acids and their derivatives—nucleotides, sugars and derivatives—carnitines—vitamin B/related metabolites and finally amines. Out of all metabolites measured, nucleotide D-ribose 5-phosphate, amino acid ornithine and multiple members of the urea cycle were the most abundant in these EVs [165, 169]. Metabolite profiles have also been successfully assessed for EVs from femoral head tissue with osteonecrosis [170], serum EVs from patients with bipolar disorder [171] and pancreatic cancer patients [172], plasma EVs from mild and severe acute pancreatitis [169], but also from CAFs [173] and adenocarcinoma cell line PANC1 [174]. Importantly, Vallabhaneni et al. confirmed that EVs deriving from mesenchymal stromal cells contain metabolites with high abundance of lactic acid and glutamic acid [175] (Fig. 4).

## Clinical use of metabolic inhibitors

Until now, no metabolic drugs have been clinically approved yet for MM, however several of these drugs are in use for other cancer types including hematological malignancies such as leukemia [176]. 6-Mercaptopurine, which targets phosphoribosyl pyrophosphate aminotransferase during purine synthesis, is currently used to treat patients with ALL, while both enasidenib and ivosidenib affect 2-hydroxyglutarate synthesis (targeting IDH2 and IDH1) and are both approved for acute myeloid leukemia (AML) [176, 177]. l-asparaginase, which hydrolyzes l-asparagine to l-aspartic acid, is FDA-approved for ALL since the 1960s.

Other small molecules affecting metabolism are in clinical trials for both solid and hematological cancers. Currently, CPI-613 (targeting oxidative phosphorylation, NCT04217317, NCT03793140), IM156 (targeting oxidative phosphorylation, NCT03272256), and AG-270 (targeting MAT2A, NCT03435250) are tested for treatment of lymphoma, while CPI-613 (NCT03504423), and IACS-010759 (targeting oxidative phosphorylation, NCT02882321) are tested in AML patients. Other metabolic inhibitors such as AZD-3965 (NCT01791595), which affects lactate transporter MCT1, and Epacadostat (targeting indoleamine 2,3 dioxygenase-1) are also currently in clinical trial for lymphoma treatment (NCT03322384) [176, 177]. The IDH1 inhibitors olutasidenib and LY3410738 are being evaluated for AML (NCT02719574) and chronic myelomonocytic leukemia (CMML)/AML treatment, respectively (NCT04603001) [176]. KPT-9274, affecting NAD production, is currently in phase I clinical trial for non-hodgkin lymphoma patients (NCT02702492, NCT04281420) [178].

In MM, CB-839, a glutaminase inhibitor, has been successfully tested in vitro and is currently in phase I clinical trial for treatment of MM, combined with carfilzomib and dexamethasone. Preliminary results are expected by the end of 2023 (NCT03798678) [105, 176]. Importantly, metformin is currently examined in several combinations strategies in clinical trials for myeloma. For RRMM patients, metformin is combined with either ritonavir, dexamethasone or as part of a triple drug therapy with bortezomib and nelfinavir (NCT04850846, NCT03829020) [179]. CB-1158 is in phase I/II for treatment of MM, where it is combined with dexamethasone (NCT03837509) [176].

## Conclusions and future perspectives

Metabolic alterations induced by the BM microenvironment contribute greatly to MM survival. Over the last decade, several metabolic alterations have been identified in MM that facilitate disease progression. These changes in metabolic pathways are induced by both the MM cells, as well as the surrounding BM microenvironment, wherein the hypoxic niche plays an important role. Similar to other cancers, glucose and glutamine are two of the most studied metabolites in MM. Both their transporter-mediated uptake and enzymatic conversions have been targeted in MM, with promising results.

To better understand how the supportive BM microenvironment affects MM metabolism and vice versa, we should focus on how to improve the current in vitro culture models to better resemble the natural environment. As hypoxia greatly affects metabolic pathways, and the natural microenvironment is hypoxic, all studies exploring metabolic alterations in myeloma should also investigate whether and how hypoxia affects this metabolic target. A potential limiting factor of metabolic inhibitors are their off-target effects. Many metabolic pathways are not only key for cancer proliferation, but also for the function and survival of healthy cell types. Trying to limit these off-target effects remains key for the future of metabolic inhibitors as a cancer treatment strategy. A possible solution to specifically target cancer cells could be the encapsulation of drugs into liposomes. Shukla et al. successfully encapsulated metformin into liposomes using a drug-loaded film method for treatment of breast cancer. The authors observed increased efficacy and bioavailability [180].

Aside from metabolic inhibitors and genetic approaches, metabolic pathways can also be targeted by amino acid-low diets, which have been shown to affect proliferation of other cancer cell types. Lysine deprivation completely blocked proliferation in colon cancer cell line [181], while dietary restriction of methionine is currently in phase I clinical trials for numerous cancers including melanoma, lung and prostate cancer [182].

Although many metabolic pathways have been explored in MM and other cancer cell types, there are still other metabolic targets in need of investigation. Not much is known about how the folate pathway is affected in myeloma, nor how the microenvironment influences folate production. Moreover, a new interesting approach assessing the metabolite profile of exosomes could results in a better understanding of how the MM cells and other cell types in the microenvironment communicate with each other through the metabolite cargo in their exosomes. Several researchers have hinted at the presence of metabolites in exosomes, but no profiling study has been done in myeloma yet.

Altogether, targeting metabolic pathways in myeloma remains a promising approach to block myeloma cell proliferation and progression, but further research is necessary to accommodate for the complex interplay that affects cell metabolism.

## Data Availability

Data sharing is not applicable to this article as no datasets were generated or analysed during the current study.

## Abbreviations

10-TPP: Decyl-triphenylphosphonium
2-DG: 2-Deoxyglucose
ADMA: Asymmetric dimethylarginine
ADP: Adenosine diphosphate
ALL: Acute lymphatic leukemia
AML: Acute myeloid leukemia
ATP: Adosine triphosphate
BM: Bone marrow
BMSC: Bone marrow stromal cell
CAF: Cancer-associated fibroblast
CMML: Chronic myelomonocytic leukemia
DHA: Docosahexaenoic acid
ECAR: Extracellular acidification rate
EHA: Eicosapentaenoic acid
EVs: Extracellular vesicles
FABP: Fatty acid binding protein
FATP: Fatty acid transport protein
FBPase: Fructose-2,6-biphosphatase
G6PD: Glucose-6-phosphate dehydrogenase
GLUT: Glucose transporter
HIF-1α: Hypoxia-inducible factor 1α
HK: Hexokinase
HRE: Hypoxia response element
ICAM5: Intercellular adhesion molecule 5
JAK: Janus activated kinase
LDHA: Lactate dehydrogenase A
lncRNA: Long non-coding RNA
MCT: Monocarboxylate transporter
MGUS: Monoclonal gammopathy of undetermined significance
MM: Multiple myeloma
NDMM: Newly diagnosed MM
OCR: Oxygen consumption rate
PADI2: Peptidyl arginine deiminase 2
PDIA3P: Protein disulfide isomerase family A member 3 pseudogene
PDK1: Pyruvate dehydrogenase kinase 1
PEP: 1,3-Biphosphophosphoenolpyruvate
PFK: Phosphofructokinase
PFK2: 6-Phosphofructo-2-kinase
PI3K: Phosphoinositide 3-kinase
PKM2: Pyruvate kinase M2
PPP: Pentose phosphate pathway
PRL-3: Phosphatase of regenerating liver 3
PUFA: Polyunsaturated fatty acids
PYCR: Pyrroline-5-carboxylate reductase
RRMM: Relapsed/refractory MM
shRNA: Short hairpin ribonucleic acid
SLC: Solute carrier family
SMM: Smoldering myeloma
STAT3: Signal transducer and activator of transcription 3
STOML2: Stomatin-like protein 2
TCA: Tricarboxylic acid
VCd: Velcade^®^ (bortezomib) Cyclophosphamide dexamethasone
VLA-4: Very late antigen 4
VRd: Velcade^®^ (bortezomib) Revlimid^®^ (lenalidomide) dexamethasone
VTd: Velcade^®^ (bortezomib) Thalidomide dexamethasone
